# Exposure to volatile organic compounds and sarcopenia risk in US adults based on NHANES

**DOI:** 10.1038/s41598-025-11628-0

**Published:** 2025-07-15

**Authors:** Tian-Zhen Qu, Yue Zhang, Qing-Yun Huang, Xing-Lan Chen, Ye Zhu

**Affiliations:** 1 Department of General Practice, Jiangyin People’s Hospital, Shoushan Road 163, Wuxi, 214400 China; 2 Department of Cardiology, Charité – Universitätsmedizin Berlin, Charitéplatz 1, 10115 Berlin, Germany; 3 Department of Clinical Medicine, Nanjing Medical University, Longmian Avenue 101, Nanjing, 210000 China; 4 Department of gerontology, Geriatric Hospital Affiliated to Nanjing Medical University, Luojia Road 30, Nanjing, 210000 China; 5 Department of Medical Laboratory, Wuxi Huishan District Center for Disease Control and Prevention, Huiyuan Road 555, Wuxi, 214174 China

**Keywords:** Sarcopenia, VOCs, MVOCs, NHANES, Environmental exposure., Epidemiology, Environmental sciences, Diseases, Medical research, Risk factors

## Abstract

This study investigates the association between volatile organic compounds (VOCs) and sarcopenia in a cohort of 3,391 U.S. participants aged 20–59 years, using NHANES data from 2011 to 2018. Various urinary metabolites of VOCs (mVOCs) were analyzed, and in multivariate logistic regression, CEMA (a metabolite of acrolein), CYMA (a metabolite of acrylonitrile), ATCA (a metabolite of cyanide), and 3,4-MHA (a metabolite of xylene) were found to have significant associations with sarcopenia. Weighted Quantile Sum (WQS) and Bayesian Kernel Machine Regression (BKMR) analyses further supported these findings, identifying ATCA as a key contributor. Subgroup analyses revealed meaningful associations between sarcopenia and mVOCs in both men and women. Additionally, mediation analysis identified high-density lipoprotein (HDL) as a partial mediator, contributing to 3.8% of the effect. These results emphasize the need for future longitudinal studies to establish causality and further explore the underlying biological mechanisms.

**Supplementary Information:**

The online version contains supplementary material available at 10.1038/s41598-025-11628-0.

## Introduction

Sarcopenia refers to the gradual reduction in both skeletal muscle strength and mass, represents a major public health issue, particularly among older adults^1^. It leads to higher risks of falls, fractures, disability, and death, which in turn impose significant socioeconomic burdens^2^. The prevalence of sarcopenia escalates with age, affecting approximately 5–13% of individuals aged 60–70 years and rising to 11–50% among those aged 80 and older^3^. The multifactorial etiology of sarcopenia involves a complex interplay of age-related changes in muscle fiber composition, neuromuscular junction integrity, hormonal regulation, and inflammatory responses^4,5^. Despite extensive research, there remains a substantial gap in understanding the role of environmental exposures, specifically to volatile organic compounds (VOCs), in the development and progression of this condition.

VOCs are a diverse group of chemicals prevalent in the environment, originating from both natural and anthropogenic sources^6^. VOCs are commonly found in indoor and outdoor air, emanating from sources such as industrial emissions, vehicle exhaust, tobacco smoke, and household products^7^. These compounds are known to have various adverse health effects, including respiratory and cardiovascular diseases, neurotoxicity, and cancer^8,9^. VOC exposure has been associated with oxidative stress and inflammatory responses, which are key contributors to muscle degradation^10,11^. Similarly, evidence suggests that air pollution, which includes VOCs, may exacerbate conditions like osteoarthritis and could potentially extend to sarcopenia due to shared inflammatory pathways^12^. Moreover, research has demonstrated the neurotoxic effects of VOCs, such as toluene, on the central nervous system, which could indirectly contribute to sarcopenia through impaired neuromuscular function^13^.Despite these findings, the relationship between VOC exposure and sarcopenia has been minimally explored.

Given the widespread presence of VOCs in the environment and their known health effects, investigating their role in sarcopenia is of significant public health importance. While previous research has provided insights into the adverse health effects of VOCs, there remains a paucity of data specifically addressing their impact on muscle health and sarcopenia risk​. Urinary metabolites of VOCs (mVOCs) serve as important biomarkers for VOC exposure due to their non-invasive nature of sample collection and the long physiological half-lives^14^. Our study extends this body of evidence by demonstrating a significant association between VOC exposure and sarcopenia by specifically analyzing the urinary metabolites of VOCs. This approach not only underscores the broader health implications of VOC exposure but also highlights the utility of mVOCs in studying environmental impacts on muscle health. By utilizing NHANES data, which provides a nationally representative sample of the U.S. population, our findings offer valuable insights that are generalizable to broader contexts. This study’s focus on mVOCs enables a precise assessment of VOC exposure and its direct association with sarcopenia, contributing to a more comprehensive understanding of environmental risk factors affecting muscle health.

To achieve this, we employed logistic regression, Weighted Quantile Sum (WQS) analysis, Bayesian Kernel Machine Regression (BKMR) analysis, and mediation analysis. These methods allowed us to assess individual and combined effects of VOCs, explore non-linear relationships, and identify potential mediating serum biomarkers, providing a comprehensive understanding of the underlying mechanisms.

## Methods

### Population

This cross-sectional study analyzed data from the National Health and Nutrition Examination Survey (NHANES) collected between 2011 and 2018, including 3,391 participants aged 20 to 59 years. Participants with missing data on key variables, such as urine creatinine, VOC exposure biomarkers, body mass index (BMI), or dual-energy X-ray absorptiometry (DXA) measurements, were excluded. The study population was selected based on the availability of data relevant to VOC exposure and sarcopenia assessment.

### Primary exposure

Participants provided urine samples without needing to follow any dietary or fasting restrictions, which were then stored at − 70 °C until analysis. Urinary mVOC levels were quantified using ultra-performance liquid chromatography paired with electrospray tandem mass spectrometry. Chromatographic separation was accomplished on an Acquity UPLC^®^ HSST3 column, utilizing 15 mM ammonium acetate and acetonitrile as the mobile phases. The eluent underwent ionization through an electrospray interface, generating negative ions which were then analyzed by the mass spectrometer. Individual analyte concentrations were calculated by comparing the relative response factors, which are the ratios of the native analyte to the stable isotope-labeled internal standard, against known reference standards. mVOCs were selected for analysis based on two criteria: (1) a detection rate of at least two-thirds (≥ 66.7%) of participants, following established practice to ensure data quality and minimize the impact of values below the detection limit^15,16^; and (2) prior toxicological and epidemiological evidence suggesting their potential relevance to oxidative stress, inflammation, metabolic disorders, or musculoskeletal health, which are mechanistically linked to sarcopenia^17,18^.

### Outcome

We assessed sarcopenia using Dual-Energy X-ray Absorptiometry (DXA) with Hologic Discovery Model A densitometers, focusing on appendicular skeletal muscle mass. Exclusion criteria for DXA scans included pregnancy, weight over 300 pounds (136 kg), height over 6′5″, recent barium contrast exposure within the last 7 days, or nuclear medicine procedures conducted within 3 days. Scans used the Hologic software. The sarcopenia index was determined by dividing total appendicular skeletal muscle mass by BMI (kg/m²). We applied sex-specific thresholds following the recommendations from the National Institutes of Health (FNIH), with values of 0.789 for men and 0.512 for women^19^.

### Covariates

Based on previous epidemiological studies assessing potential risk factors for sarcopenia^20,21^, the analysis accounted for several covariates, including demographic variables (sex, age, race, education level, and Poverty Income Ratio (PIR)), lifestyle characteristics (BMI, sedentary behavior, smoking habits, and alcohol use), health conditions (hypertension, diabetes), and serum concentrations of vitamin D (25OHD3 + 25OHD2). The PIR was calculated as the ratio of annual income to the poverty threshold, adjusted for family size. Participants were asked if they had consumed at least 12 alcoholic drinks in the past year to assess their drinking status. Smoking status was defined by whether an individual had smoked a minimum of 100 cigarettes in their lifetime. Diagnoses of diabetes mellitus and hypertension were based on self-reports from participants who had been diagnosed by healthcare professionals.

**Fig. 1:**
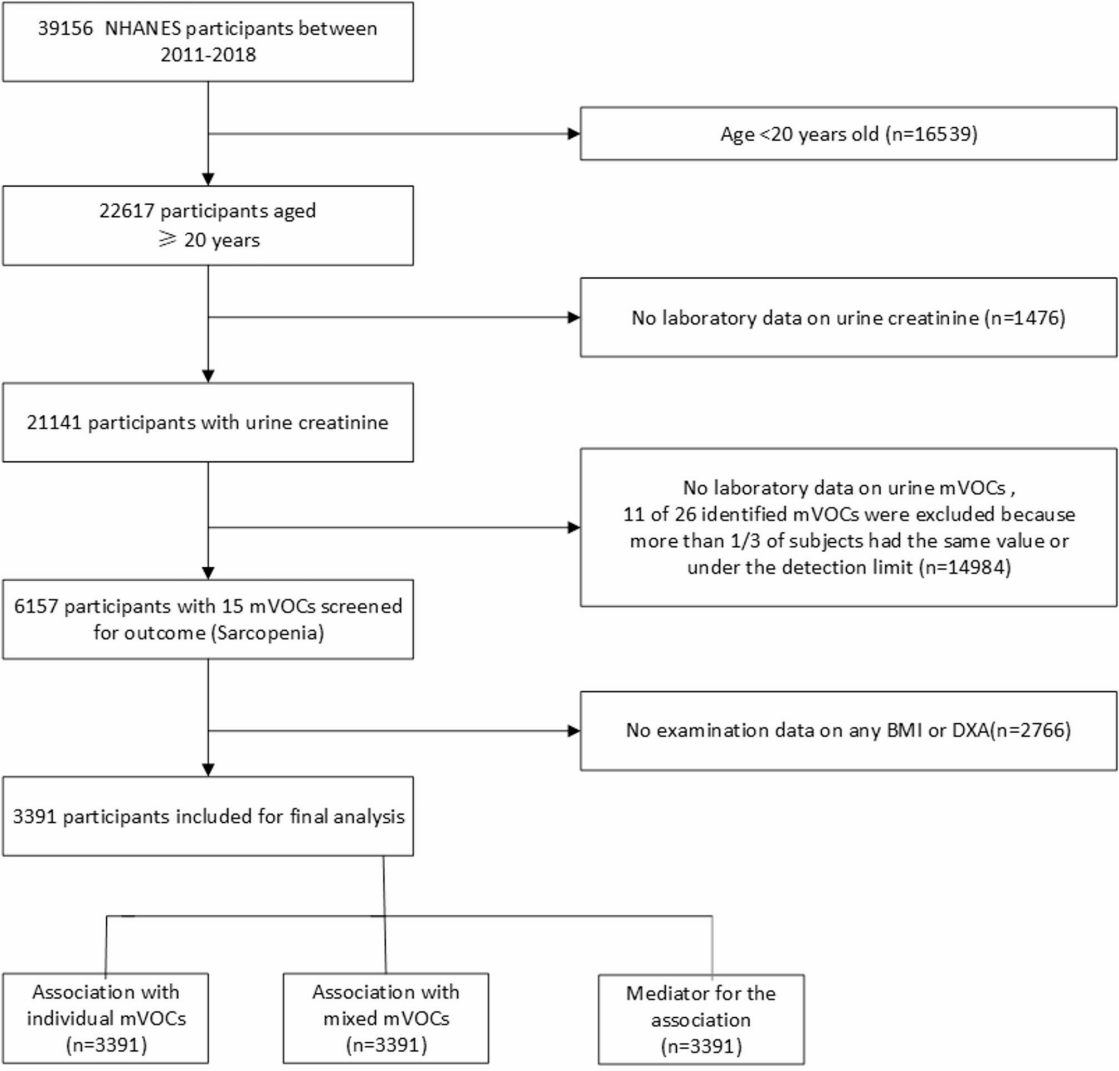
Flowchart of NHANES participants included for final analysis.Note: All three analyses (individual mVOCs, mixture, mediation) used the same cohort of 3,391 participants; no further exclusions were applied.

### Statistical analysis

Descriptive statistics were conducted on the baseline characteristics of the sarcopenia and control groups. Continuous variables, shown as means ± standard deviations, were analyzed using t-tests or Mann–Whitney U tests. Categorical variables, expressed as weighted proportions, were examined using weighted chi-square tests. Urinary mVOC concentrations were adjusted for creatinine and underwent log-transformation to achieve a normal distribution. Survey-adjusted multivariate logistic regression models were utilized to evaluate the relationship between sarcopenia and exposure to individual VOCs. We designated the control group as the first quartile (Q1). Following NHANES guidelines, we applied subsample weights to ensure the results were representative of the U.S. population in all analyses.

Second, we used a Weighted Quantile Sum (WQS) regression model to assess the impact of co-exposure to mVOCs on sarcopenia. The model grouped the various mVOCs into quartiles and calculated a weighted linear index to represent their combined effects.The WQS index was constructed using quartiles (q = 4) of mVOC concentrations, which is consistent with standard practice in NHANES-based WQS analyses and provides stable and interpretable estimates. Each mVOC’s weight reflected its contribution to the WQS index. The dataset was randomly split into a training set (40%) and a validation set (60%) during model fitting, to estimate mixture weights and reduce overfitting, following standard practice for WQS regression. Final model results were reported using the full dataset. The model was developed using the R ‘gWQS’ package, employing 1000 bootstrapped iterations.

Third, BKMR was used to analyze the combined effect of 15 mVOCs on sarcopenia, employing Bayesian methods and a Gaussian kernel function to explore nonlinear relationships and interactions. A probit regression with variable selection was applied for the binary outcome (sarcopenia: yes/no). Hierarchical clustering was used to analyze the 15 mVOCs, grouping them based on similarities in their characteristics. Supplementary Figure S1 evaluates the optimal number of clusters for mVOCs using the Calinski-Harabasz index and silhouette coefficient. Both metrics indicate that higher values signify better clustering performance. The Calinski-Harabasz index shows that two clusters provide a high ratio of between-cluster to within-cluster dispersion, indicating strong clustering effectiveness. Similarly, the silhouette coefficient confirms that two clusters offer the best consistency, with the highest similarity within clusters compared to other clusters. Therefore, two clusters were chosen for this analysis. The BKMR analysis results were utilized for hierarchical variable selection. Group posterior inclusion probability (groupPIP) and conditional posterior inclusion probability (condPIP) were used to reflect the inclusion probabilities of groups and individual chemicals within those groups, respectively, ranking the significance of each exposure. Model estimates were derived after 10,000 iterations.

To explore whether potential intermediaries mediate the impact of mVOC mixtures on sarcopenia prevalence, we conducted mediation analysis using the ‘MEDIATION’ package in R, following established methodologies. The hypothesis tested was that the link between mVOCs and sarcopenia is mediated by certain intermediaries. The total effects (TE) of mVOCs were broken down into direct effects (DE) and indirect effects (IE) on sarcopenia. The proportion of IE relative to TE provides insight into the mediators’ effectiveness.

For addressing missing data in research, mode imputation is used for categorical variables, and mean imputation is used for continuous variables. Studies have shown that when the proportion of missing data is below 10%, simple imputation methods are generally convenient and acceptable, despite potential biases^22^. All statistical analyses were conducted using R software (version 4.4.1; https://www.R-project.org/), with significance set at a P-value below 0.05.

#### Ethics approval and consent to participate

All procedures involving human participants were conducted in accordance with the Declaration of Helsinki. The study protocol was approved by the National Center for Health Statistics (NCHS) Research Ethics Review Board, and written informed consent was obtained from all participants prior to their participation in the survey. Additional details can be found on the official NHANES website.

## Results

### Participant selection

The initial sample comprised 39,156 NHANES participants from 2011 to 2018. After excluding 16,539 individuals who were under 20 years old, 22,617 participants aged 20 years or older remained. Among these, 1,476 participants were excluded due to missing laboratory data on urine creatinine, leaving 21,141 participants eligible for further analysis.

Out of the 21,141 participants with urine creatinine data, 14,984 were excluded due to the absence of laboratory data on urine mVOCs or because 11 out of 26 identified mVOCs had values below the detection limit or were consistent across more than one-third of subjects. This left 6,157 participants who were screened for sarcopenia outcomes. A further 2,766 participants were excluded due to the lack of examination data on BMI or DXA measurements. As a result, 3,391 participants were incorporated into the final analysis (Fig. 1). Supplementary Table S1 presents the parent VOCs along with their corresponding mVOC abbreviations.

### Baseline demographic and clinical characteristics

Participants with sarcopenia were older (42.28 vs. 37.05 years, *p* < 0.001) and had a lower BMI (34.16 vs. 28.03 kg/m², *p* < 0.001) compared to those without sarcopenia. Significant differences were observed in race/ethnicity, education, and PIR. Diabetes (14.98% vs. 6.28%, *p* < 0.001) and hypertension (29.48% vs. 21.09%, *p* = 0.002) were more prevalent in the sarcopenia group. They also had lower serum vitamin D levels (56.68 vs. 60.12 nmol/L, *p* = 0.022) and consumed less alcohol (59.66% vs. 71.19%, *p* < 0.001) (Table 1).

### Relationship between individual mVOCs and sarcopenia

The association of individual mVOCs with sarcopenia was evaluated using a series of models. In the unadjusted model (Model 1), several mVOCs (ATCA, CYMA and CEMA) showed varying degrees of association with sarcopenia. After controlling for sex and age in Model 2, and further controlling for all covariates—including race, education level, PIR, smoking habits, BMI, physical activity, diabetes, hypertension, alcohol intake, and serum vitamin D levels—in Model 3, the associations became clearer and more defined.

**Fig. 2:**
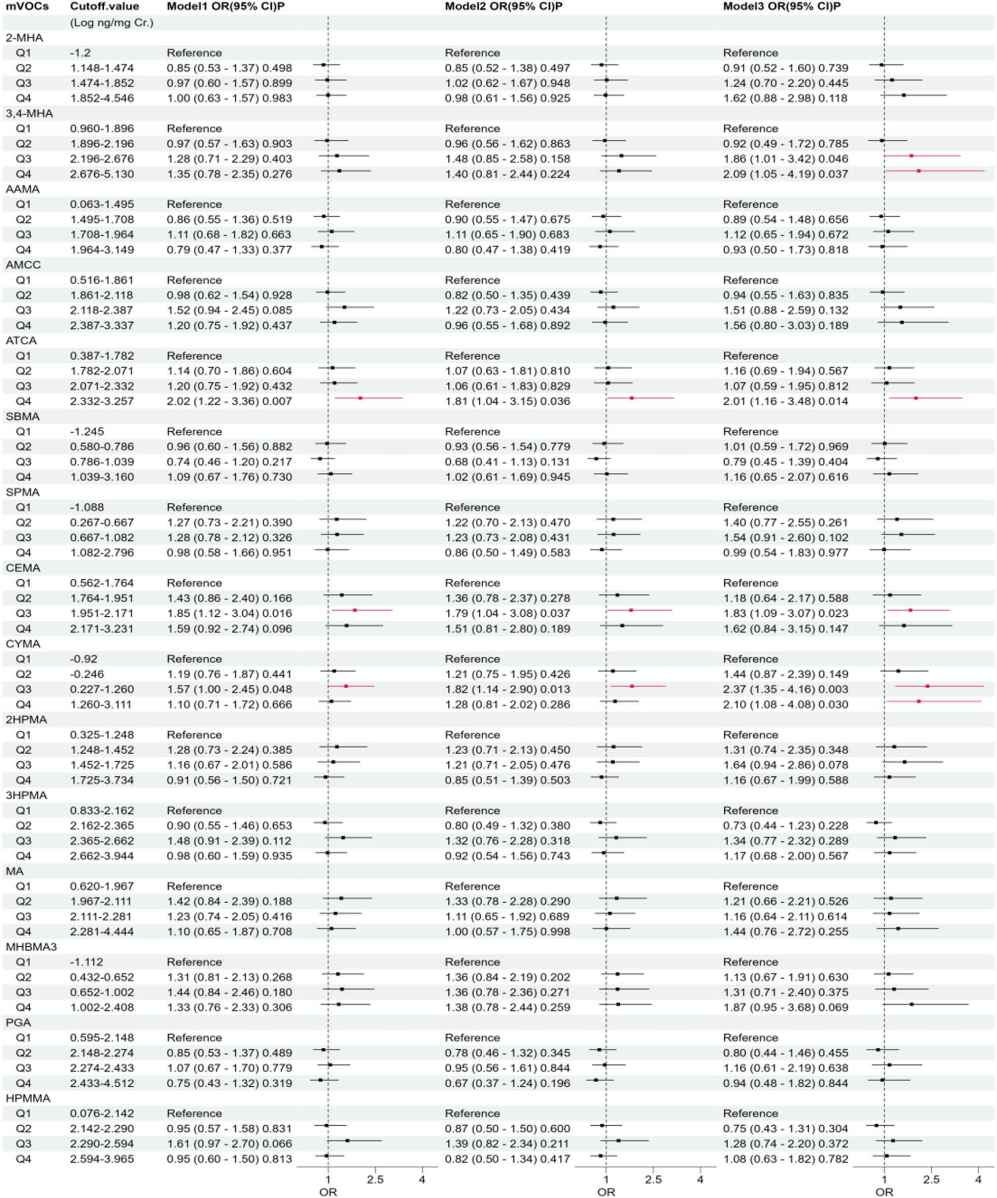
Forest plot depicting the association of individual mVOCs with sarcopenia. The odds ratios (ORs) and 95% confidence intervals (CIs) for sarcopenia are shown across quartiles of mVOC concentrations in three models. Model 1 = multivariable logistic regression model includes no adjustments. Model 2 is adjusted for age and sex. Model 3 is fully adjusted for age, sex, race/ethnicity, education level, poverty income ratio (PIR), smoking status, body mass index, physical activity, diabetes, hypertension, alcohol consumption, and serum vitamin D levels. The red lines indicate statistically significant associations (*p* < 0.05).

The forest plot (Fig. 2) highlights specific mVOCs that remained significantly associated with sarcopenia across the models. Notably, higher quartiles of exposure to certain mVOCs, such as ATCA, 3-4MHA, CYMA and CEMA, showed increased odds of sarcopenia in the fully adjusted model (Model 3). These findings suggest that exposure to higher levels of these mVOCs were linked with an elevated risk of sarcopenia, even after accounting for potential confounding factors. The associations were strongest and most consistent for ATCA and 3-4MHA, with significant odds ratios across multiple models.

The adjusted logistic regression model revealed several significant associations between mVOCs and the risk of sarcopenia across different subgroups (Supplementary Table S2):In the female subgroup, 2-MHA (Q4), 3,4-MHA (Q4), and CEMA (Q3) showed a positive association with an elevated risk of sarcopenia. Conversely, in the male subgroup, CYMA (Q2, Q3) and PGA (Q2) showed a positive association with sarcopenia risk.For the younger subgroup (age < 40 years), the model identified positive associations with sarcopenia for ACTA (Q2, Q3), CEMA (Q2, Q3), CYMA (Q3, Q4), MA (Q4), and MHBMA3 (Q3). In the elderly subgroup (age ≥ 40 years), significant associations were found with 3,4-MHA (Q3, Q4) and CYMA (Q3).Additionally, among overall population with a BMI ≥ 25, 2-MHA (Q4), 3,4-MHA (Q4), ATCA (Q4), CYMA (Q3, Q4), and MA (Q4) were linked to an increased risk of sarcopenia. Despite the small sample size in the BMI < 25 subgroup (only 24 positive samples), we conducted a logistic regression analysis for completeness. The result indicated that ATCA (Q4), SPMA (Q2, Q3, Q4), CEMA (Q3), CYMA (Q2, Q4), 2HPMA (Q3), and PGA (Q3) were linked with an increased risk of sarcopenia.

**Table 1:** Baseline characteristics of included participants.

Variable	Non-Sarcopenia (*N* = 3,123)	Sarcopenia (*n* = 268)	*p*-value
Age, years (mean ± SD)	37.05 ± 12.27	42.28 ± 12.64	< 0.001
Sex, n (%)			0.746
Female	1,565 (50.11%)	131 (48.88%)	
Male	1,558 (49.89%)	137 (51.12%)	
BMI, kg/m² (mean ± SD)	28.03 ± 6.53	34.16 ± 7.76	< 0.001
Race/Ethnicity, n(%)			< 0.001
Mexican American	1,049 (33.59%)	61 (22.76%)	
Hispanic	735 (23.54%)	16 (5.97%)	
Non-Hispanic White	418 (13.38%)	97 (36.19%)	
Non-Hispanic Black	318 (10.18%)	46 (17.16%)	
Other Race	603 (19.31%)	48 (17.91%)	
Education level, n (%)			< 0.001
Less than 9th grade	157 (5.63%)	42 (18.42%)	
9–12th grade or equivalent	790 (28.31%)	75 (32.89%)	
College or above	1,844 (66.07%)	111 (48.68%)	
(Missing)	332	40	
PIR, n (%)			0.015
≤ 1	626 (22.01%)	56 (23.63%)	
1–3	1,139 (40.05%)	113 (47.68%)	
≥ 3	1,079 (37.94%)	68 (28.69%)	
(Missing)	279	31	
Sedentary activity, min (mean ± SD)	368.44 ± 203.16	348.66 ± 212.08	0.143
(Missing)	9	0	
Smoking, n(%)			0.773
No	1,919 (63.13%)	165 (62.03%)	
Yes	1,121 (36.88%)	101 (37.97%)	
(Missing)	83	2	
Had at least 12 alcohol drinks/1 year? n(%)			< 0.001
No	820 (28.81%)	96 (40.34%)	
Yes	2,026 (71.19%)	142 (59.66%)	
(Missing)	277	30	
Diabetes, n (%)			< 0.001
No	2,925 (93.72%)	227 (85.02%)	
Yes	196 (6.28%)	40 (14.98%)	
(Missing)	2	1	
Hypertension, n (%)			0.002
No	2,462 (78.91%)	189 (70.52%)	
Yes	658 (21.09%)	79 (29.48%)	
(Missing)	3	0	
Serum vitamin D, nmol/L (mean ± SD)	60.12 ± 25.20	56.68 ± 22.90	0.022
(Missing)	140	8	

BMI, body mass index; SD, standard deviation; PIR, poverty income ratio.

### WQS analysis

**Fig. 3:**
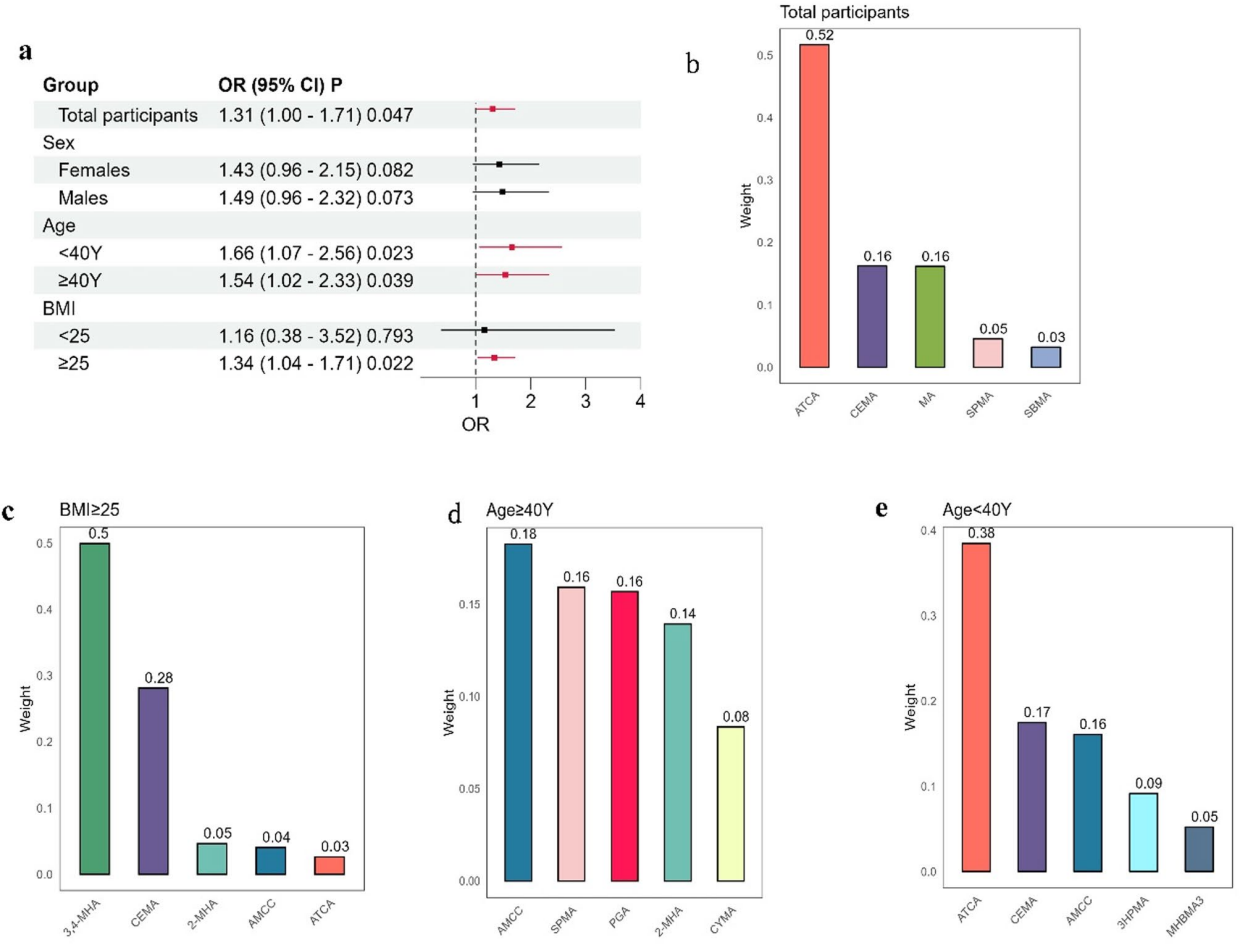
Weighted Quantile Sum (WQS) analysis of the association between mVOCs and sarcopenia. (**a**) Forest plot depicting the overall odds ratio (OR) and 95% confidence intervals (CIs) for the association, stratified by sex, age, and BMI. (**b**-**e**) Bar charts showing the weights of individual mVOCs in the WQS index for the total participants (**b**), participants with BMI ≥ 25 (**c**), participants aged ≥ 40 years (**d**), and participants aged < 40 years (**e**). The mVOCs with a weighted value ranking top 5 were shown. The WQS regression model was adjusted for age, sex, race/ethnicity, education level, poverty income ratio (PIR), smoking status, body mass index, physical activity, diabetes, hypertension, alcohol consumption, and serum vitamin D levels.

WQS analysis demonstrated a notable correlation between mVOCs and sarcopenia after adjusting for all covariates. The overall odds ratio (OR) for all participants was 1.31 (95% CI: 1.00-1.71, *p* = 0.047) (Fig. 3a). Subgroup analyses identified significant associations in participants younger than 40 years (OR: 1.66, 95% CI: 1.07–2.56, *p* = 0.023), those older than 40 years (OR: 1.54, 95% CI: 1.02–2.33, *p* = 0.039), and individuals with a BMI of 25 or more (OR: 1.34, 95% CI: 1.04–1.71, *p* = 0.022).

The WQS index weights indicated that ATCA had the highest contribution to the association with sarcopenia across all groups. Specifically, ATCA had a weight of 0.52 in the total participants (Fig. 3b) and 0.38 in participants under 40 years old (Fig. 3e). In the BMI ≥ 25 group, 3,4-MHA had the largest weight at 0.5 (Fig. 3c), while AMCC contributed the highest in the age ≥ 40 years group, with a weight of 0.18 (Fig. 3d).

**Fig. 4:**
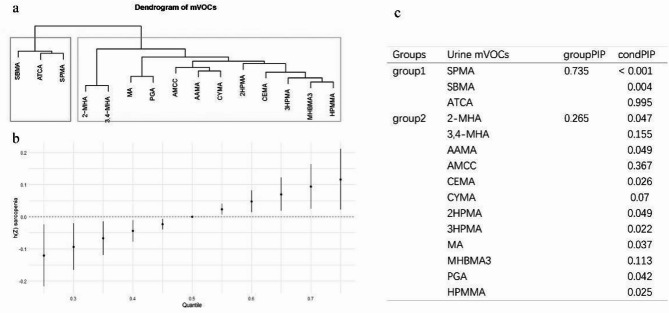
Results of the cluster and Bayesian Kernel Machine Regression (BKMR) analysis of mVOCs and sarcopenia. (**a**) Dendrogram from the cluster analysis, dividing mVOCs into two distinct clusters. (**b**) The joint effect (95% CI) of mVOCs on sarcopenia when all mVOCs at particular percentiles were compared to all mVOCs at their 50th percentile. In the BKMR model, h(Z) represents the change in the log odds of sarcopenia associated with varying levels of mixed mVOC exposure. (**c**) Table displaying the groups of mVOCs with their group posterior inclusion probability (groupPIP) and conditional posterior inclusion probability (condPIP). The BKMR model was adjusted for age, sex, race/ethnicity, education level, poverty income ratio (PIR), smoking status, body mass index, physical activity, diabetes, hypertension, alcohol consumption, and serum vitamin D levels. mVOCs, metabolites of volatile organic compounds.

### BKMR analysis

The BKMR analysis, following the initial cluster analysis, revealed significant associations between certain mVOCs and sarcopenia risk. The dendrogram (Fig. 4a) grouped the mVOCs into two distinct clusters: Cluster 1 (SPMA, SBMA, and ATCA) and Cluster 2 (the remaining mVOCs). This clustering highlights the similarities in the behavior of mVOCs within each group.

The BKMR analysis, adjusted for all covariates, demonstrated that higher quantiles of mVOC exposure were linked to an elevated risk of developing sarcopenia (Fig. 4b). The point estimate plot shows a positive trend, indicating that as the quantiles of exposure to these mVOCs increase, the estimated risk of sarcopenia also increases.

Additionally, the table in Fig. 4C presents the group posterior inclusion probability (groupPIP) and conditional posterior inclusion probability (condPIP) for each mVOC group. Cluster 1 had a groupPIP of 0.735 (*p* < 0.001), indicating a strong association with sarcopenia, while Cluster 2 had a groupPIP of 0.265. These findings suggest that the mVOCs in Cluster 1, particularly ATCA, have a more substantial impact on sarcopenia risk compared to those in Cluster 2.

### Mediated analysis

**Fig. 5:**
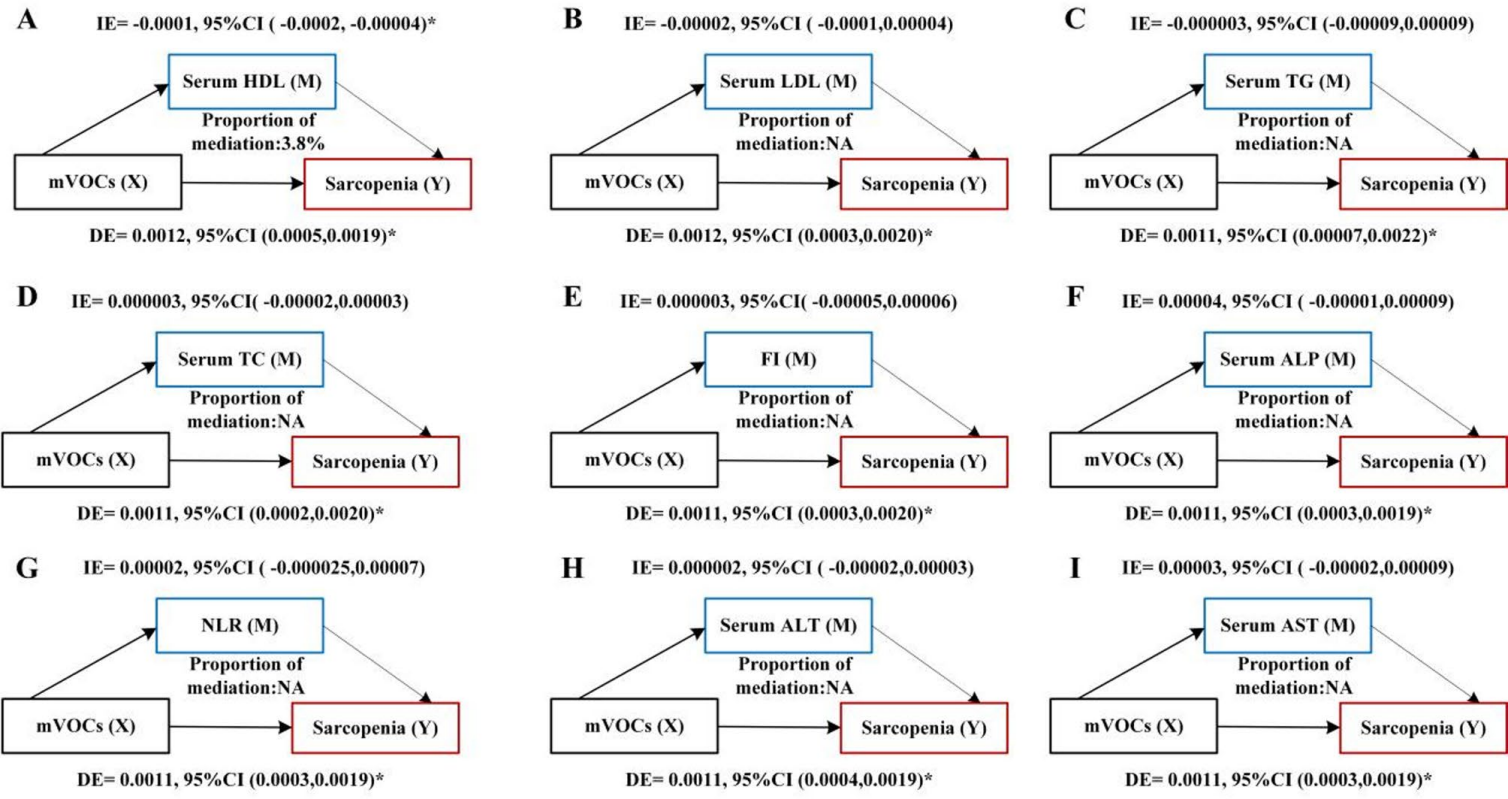
Mediation analysis of the indirect effects of various serum biomarkers on the relationship between mVOCs and sarcopenia. Each panel (**a**-**f**) shows the mediation pathways for different serum biomarkers. (**a**) Serum High-Density Lipoprotein (Serum HDL), (**b**) Serum Low-Density Lipoprotein (LDL), (**c**) Serum Triglycerides (TG), (**d**) Serum Total Cholesterol (TC), (**e**) Fasting Insulin (FI), (**f**) Serum Alkaline Phosphatase (ALP). Models were adjusted for age, sex, race/ethnicity, education level, poverty income ratio (PIR), smoking status, body mass index, physical activity, diabetes, hypertension, alcohol consumption, and serum vitamin D levels. The indirect effects (IE), direct effects (DE) and 95% confidence intervals (CIs) are provided, indicating the proportion of mediation for each biomarker. Proportion of mediation = IE/(DE + IE); NA, not available. *P < 0.05.

The mediated analysis, adjusted for all covariates, revealed that serum HDL significantly mediated the association between mVOCs and sarcopenia. Specifically, serum HDL exhibited an IE of −0.0001 (95% CI: −0.0002 to −0.00004), contributing 3.8% to the TE (Fig. 5a). However, other serum markers (Fig. 5b-f) did not show significant mediation in this association.

## Discussion

The primary results from this research indicate a notable link between VOC exposure and sarcopenia, with a particular focus on urinary mVOCs as biomarkers.Using data from NHANES 2011–2018, our multivariate logistic regression analysis identified a positive relationship between sarcopenia and exposure to various mVOCs, including CEMA, CYMA, ATCA, and 3,4-MHA. These mVOCs are metabolites of the parent compounds acrolein, acrylonitrile, cyanide, and xylene, respectively. Urine ATCA, a cyanide metabolite, was highlighted as the most influential factor in both the BKMR and WQS analyses, which demonstrated a positive association between co-exposure to VOCs and sarcopenia.

Cyanide, a common VOC, is widely utilized in industries such as mining, chemicals, electroplating, and plastics production^23^. When cyanide enters the body, it quickly converts to hydrocyanic acid (HCN). Most of it is detoxified in the liver by sulfurtransferases, turning cyanide into thiocyanate, which is then excreted. However, some cyanide combines with cysteine to form a compound that is further hydrolyzed to ATCA^24^.When inhaled or ingested, cyanide free radicals bind to the iron in cytochrome oxidase in the mitochondria. This prevents normal cell respiration, leading to tissue hypoxia and suffocation^25^. Mitochondria within skeletal muscle fibers play a crucial role in ATP production. Beyond generating energy, they also regulate calcium levels and control reactive oxygen species (ROS) production, both of which are essential for muscle function and adaptation^26^. When mitochondria become dysfunctional, it can result in increased oxidative stress and impaired muscle function, ultimately contributing to muscle wasting^27^. Animal models also show this connection clearly. In the mtDNA mutant mouse model, mutations disrupt mitochondrial energy production and ATP balance, causing skeletal muscle cell death and sarcopenia, and mice with Pgc-1α knockout show similar results^28,29^. While the link between cyanide exposure and sarcopenia is biologically plausible, additional study is required to confirm this relationship and explore the underlying mechanisms.

The mediation analysis indicated that HDL was the sole mediator in the link between multiple VOC co-exposures and sarcopenia, contributing to 3.8% of the mediating effect. Interestingly, we observe that the indirect effect (IE) is negative, while the direct effect (DE) is positive, indicating a possible suppression effect^30^. This means that mVOCs may increase the risk of sarcopenia by lowering HDL levels (resulting in a negative IE). HDL, commonly known as “good” cholesterol, typically protects muscle health through its anti-inflammatory and antioxidant properties^31^.When exposure to mVOCs reduces HDL levels, it diminishes HDL’s protective effect on muscles, indirectly raising the risk of sarcopenia. Thus, while mVOCs lower HDL levels and increase the risk of sarcopenia through this indirect path, this negative indirect effect might partly obscure the positive direct effect of mVOCs on sarcopenia.In other words, when the influence of HDL is controlled, the direct effect of mVOCs on sarcopenia becomes more apparent and significant. While exploring the relationship between mVOCs exposure and sarcopenia, it is possible that more significant positive mechanisms remain unexamined. Studies have shown that sarcopenia patients often present with elevated levels of inflammatory mediators, including IL-6, TNF-α, and CRP^32^. Unfortunately, the NHANES database from 2011 to 2018 does not include data on these inflammatory markers. Future studies could benefit from incorporating this information to provide a more comprehensive understanding.

In female, 2-MHA, 3,4-MHA, and CEMA were positively associated with sarcopenia, whereas in male, urinary CYMA and PGA showed similar associations. The parent VOCs for 2-MHA, 3,4-MHA, and CEMA are xylene and acrolein, commonly found in household cleaners and cooking fumes, which women are more likely to encounter^33^. Conversely, CYMA and PGA, derived from acrylonitrile, ethylbenzene, and styrene, are prevalent in industrial settings, exposing male workers to higher concentrations^34^. Alongside environmental exposure, alterations in the endocrine system may significantly influence the link between VOCs and sarcopenia. Studies indicate that certain VOCs can mimic or inhibit sex hormones, leading to hormonal imbalances. For instance, styrene interferes with androgen function, significantly lowering testosterone levels in exposed workers^35^. Additionally, some VOCs used in oil and gas extraction act as endocrine disruptors, altering sex hormone receptor activity^36^. Since sex hormones are crucial for muscle health, hormone replacement therapies, such as testosterone and estrogen, have been shown to improve muscle mass and performance in sarcopenia patients^37,38^. These findings suggest that VOC exposure may disrupt hormonal balance, thereby increasing the risk of sarcopenia. Moreover, within the group of individuals with a BMI of 25 or higher, we found that xylene is strongly associated with sarcopenia, likely due to its high fat solubility^39^. This observation suggests that the different fat proportions between sexes might play a role in the varying impacts of VOC exposure on muscle health. Given that women generally have a higher body fat percentage compared to men, this could lead to greater accumulation and prolonged retention of lipophilic compounds like xylene, thereby increasing their risk of sarcopenia.

In the current study, age-stratified sub-analysis revealed that in the younger subgroup, urinary levels of ACTA, CEMA, CYMA, MA, and MHBMA3 were positively correlated with an increased risk of sarcopenia. In contrast, in the elderly subgroup, increased levels of 3,4-MHA and CYMA were linked to a greater risk of sarcopenia development. This variation can be partly explained by age-related variations, VOC exposure levels, and co-existing conditions such as hypertension and diabetes^40^. Given that hypertension and diabetes elevate the likelihood of developing sarcopenia^41,42^, and considering the positive correlation between VOC exposure and both hypertension and diabetes^43,44^, it is plausible that VOCs may elevate the risk of sarcopenia by influencing blood pressure and insulin resistance. As a result, conditions like hypertension and diabetes may contribute causally to this relationship. Notably, after adjusting for multiple confounding variables, including hypertension and diabetes, the significant positive link between VOC exposure and sarcopenia remained. This suggests that VOC exposure may have a direct association with sarcopenia, independent of the effects of VOC-related hypertension and diabetes.

The findings suggest that reducing VOC exposure could be a preventative strategy against sarcopenia. Specific public health measures could include implementing stricter environmental regulations to limit industrial and vehicular emissions of VOCs, and promoting the use of low-VOC household products and building materials. Clinically, healthcare providers should consider including VOC exposure history in sarcopenia risk assessments and advising patients on protective measures such as using air purifiers and wearing masks when necessary.

### Study limitations and strengths

This work has several strengths. This is the first study to investigate the associations between individual urinary mVOCs and sarcopenia using a variety of statistical models. Our findings support epidemiological evidence on the impact of VOCs on muscle health and offer new insights for health risk assessment of environmental VOC exposure. Second, we performed a cluster analysis on mVOCs, creating two groups based on consistency metrics, which reduced the impact of high correlations among exposures. Using BKMR further enhanced our analysis by accounting for complex interactions between mVOCs and sarcopenia. Additionally, we used a representative sample of the U.S. population with a sufficient sample size.

Nevertheless, several limitations should be considered. First, the cross-sectional nature of this study restricts our ability to infer a causal relationship between mVOCs and sarcopenia. Second, the subgroup with a BMI < 25 had a relatively small sample size (24), which is below the commonly accepted threshold for statistical analysis. Lastly, we applied data imputation to address missing values. While this approach helped maintain the sample size, it may have introduced biases, potentially impacting the robustness and generalizability of our results.

## Conclusion

This cross-sectional study demonstrated that exposure to individual VOCs, such as cyanide, acrolein, acrylonitrile, and xylene, as well as co-exposure to multiple VOCs, was strongly associated to an increased risk of sarcopenia in the U.S. population. These findings underscore the potential health impacts of environmental VOC exposure on sarcopenia risk. Future research should focus on establishing causality through longitudinal studies, expanding sample sizes, particularly for underrepresented subgroups, and exploring the biological mechanisms underlying these associations. Additionally, validating biomarkers for VOC exposure and sarcopenia will be crucial. Interventions targeting VOC exposure reduction could provide valuable public health benefits in mitigating sarcopenia risk.

## Electronic supplementary material

Below is the link to the electronic supplementary material.

Supplementary Material 1

Supplementary Material 2

Supplementary Material 3

## Data Availability

Publicly available datasets were analyzed in this study. This data can be found here: https://wwwn.cdc.gov/nchs/nhanes/.
