# Supplementary material for: Exposure to volatile organic compounds and sarcopenia risk in US adults based on NHANES
Source: Sci Rep. 2025 Jul 15;15:25480. doi: 10.1038/s41598-025-11628-0 (PMC12264073; doi:10.1038/s41598-025-11628-0)
Supplement: Supplementary file 1 — Supplementary Material 1 [file 41598_2025_11628_MOESM1_ESM.docx]

| mVOCs | MALE | FEMALE | ≥40y | <40y | BMI≥25 | BMI<25 |
| --- | --- | --- | --- | --- | --- | --- |
| 2-MHA |  |  |  |  |  |  |
| Q1 | Reference | Reference | Reference | Reference | Reference | Reference |
| Q2 | 0.58(0.23-1.43)0.229 | 1.25(0.57-2.75)0.566 | 0.71(0.28-1.75)0.444 | 1.11(0.56-2.23)0.753 | 0.91(0.51-1.62)0.745 | 0.97(0.24-3.90)0.967 |
| Q3 | 0.73(0.39-1.38)0.326 | 2.13(0.91-4.97)0.080 | 1.83(0.71-4.68)0.205 | 0.77(0.36-1.68)0.508 | 1.29(0.73-2.29)0.371 | 0.98(0.20-4.73)0.979 |
| Q4 | 1.10(0.49-2.43)0.817 | 2.51(1.21-5.21)0.015 | 2.44(0.99-6.03)0.053 | 0.96(0.51-1.81)0.906 | 1.88(1.01-3.53)0.048 | 0.12(0.01-1.11)0.061 |
| 3,4-MHA |  |  |  |  |  |  |
| Q1 | Reference | Reference | Reference | Reference | Reference | Reference |
| Q2 | 0.70(0.30-1.63)0.405 | 1.06(0.44-2.57)0.886 | 1.17(0.47-2.96)0.728 | 0.80(0.37-1.72)0.562 | 0.90(0.48-1.69)0.743 | 1.56(0.37-6.55)0.534 |
| Q3 | 1.68(0.84-3.36)0.142 | 2.12(0.82-5.49)0.118 | 4.02(1.47-10.99)0.008 | 0.82(0.41-1.65)0.571 | 1.73(0.94-3.19)0.079 | 3.93(0.94-16.49)0.061 |
| Q4 | 1.52(0.57-4.08)0.392 | 2.84(1.20-6.75)0.019 | 3.30(1.12-9.71)0.031 | 1.46(0.72-2.95)0.284 | 2.30(1.11-4.76)0.025 | 0.46(0.06-3.75)0.460 |
| AAMA |  |  |  |  |  |  |
| Q1 | Reference | Reference | Reference | Reference | Reference | Reference |
| Q2 | 0.96(0.39-2.34)0.924 | 0.77(0.36-1.64)0.488 | 1.03(0.50-2.15)0.926 | 0.68(0.39-1.22)0.191 | 0.88(0.51-1.52)0.646 | 1.39(0.28-6.94)0.681 |
| Q3 | 1.22(0.56-2.65)0.615 | 0.98(0.45-2.13)0.963 | 1.30(0.52-3.25)0.574 | 0.83(0.51-1.36)0.454 | 1.20(0.69-2.09)0.516 | 0.42(0.08-2.16)0.294 |
| Q4 | 1.18(0.48-2.89)0.709 | 0.79(0.34-1.82)0.566 | 0.71(0.29-1.76)0.450 | 1.14(0.48-2.71)0.762 | 1.07(0.57-2.02)0.821 | 0.31(0.03-3.32)0.324 |
| AMCC |  |  |  |  |  |  |
| Q1 | Reference | Reference | Reference | Reference | Reference | Reference |
| Q2 | 1.48(0.75-2.92)0.257 | 0.50(0.22-1.14)0.096 | 0.60(0.23-1.58)0.295 | 1.46(0.80-2.67)0.207 | 0.87(0.49-1.56)0.642 | 2.05(0.39-10.88)0.389 |
| Q3 | 1.73(0.72-4.17)0.217 | 1.23(0.56-2.69)0.597 | 1.42(0.62-3.26)0.393 | 1.83(0.99-3.41)0.056 | 1.42(0.80-2.53)0.229 | 2.40(0.86-6.76)0.094 |
| Q4 | 1.33(0.48-3.69)0.573 | 1.62(0.78-3.34)0.190 | 1.46(0.55-3.89)0.436 | 1.76(0.73-4.20)0.200 | 1.57(0.78-3.16)0.200 | 0.96(0.09-9.65)0.968 |
| ATCA |  |  |  |  |  |  |
| Q1 | Reference | Reference | Reference | Reference | Reference | Reference |
| Q2 | 1.31(0.74-2.30)0.344 | 0.81(0.19-3.51)0.778 | 0.67(0.30-1.47)0.305 | 2.15(1.04-4.45)0.040 | 1.16(0.68-1.97)0.570 | 3.50(0.43-28.26)0.234 |
| Q3 | 1.23(0.54-2.79)0.612 | 0.99(0.25-4.02)0.992 | 0.53(0.20-1.39)0.190 | 2.50(1.05-5.96)0.039 | 1.06(0.57-1.96)0.859 | 6.08(0.72-51.32)0.095 |
| Q4 | 0.99(0.46-2.11)0.969 | 2.65(0.64-10.95)0.173 | 1.80(0.78-4.15)0.163 | 2.54(0.88-7.34)0.083 | 1.99(1.11-3.57)0.022 | 7.91(1.32-47.33)0.025 |
| SBMA |  |  |  |  |  |  |
| Q1 | Reference | Reference | Reference | Reference | Reference | Reference |
| Q2 | 1.21(0.63-2.32)0.554 | 0.73(0.31-1.73)0.465 | 0.99(0.47-2.06)0.972 | 1.05(0.59-1.87)0.858 | 0.98(0.56-1.72)0.945 | 2.29(0.44-11.79)0.314 |
| Q3 | 0.61(0.27-1.34)0.208 | 0.89(0.40-1.99)0.774 | 0.77(0.37-1.62)0.482 | 0.86(0.35-2.12)0.744 | 0.78(0.44-1.39)0.386 | 2.12(0.51-8.86)0.295 |
| Q4 | 1.46(0.67-3.17)0.335 | 0.86(0.33-2.25)0.757 | 0.94(0.39-2.23)0.878 | 1.60(0.72-3.52)0.240 | 1.18(0.65-2.15)0.574 | 2.16(0.35-13.44)0.401 |
| SPMA |  |  |  |  |  |  |
| Q1 | Reference | Reference | Reference | Reference | Reference | Reference |
| Q2 | 1.17(0.54-2.52)0.688 | 1.50(0.73-3.08)0.267 | 1.17(0.58-2.38)0.649 | 1.87(0.86-4.07)0.110 | 1.38(0.75-2.51)0.289 | 1.02e7(8.36e5-1.25e8)<0.001 |
| Q3 | 1.96(0.83-4.60)0.120 | 1.11(0.50-2.43)0.796 | 1.65(0.75-3.60)0.206 | 1.49(0.67-3.29)0.319 | 1.47(0.86-2.52)0.159 | 2.08e7(5.38e6-8.05e7)<0.001 |
| Q4 | 1.33(0.56-3.16)0.517 | 0.69(0.32-1.51)0.349 | 0.93(0.39-2.19)0.858 | 1.21(0.51-2.88)0.657 | 0.87(0.45-1.69)0.671 | 2.91e7(1.11e7-7.63e7)<0.001 |
| CEMA |  |  |  |  |  |  |
| Q1 | Reference | Reference | Reference | Reference | Reference | Reference |
| Q2 | 0.89(0.36-2.21)0.800 | 1.38(0.59-3.23)0.452 | 0.78(0.28-2.20)0.636 | 2.17(1.01-4.69)0.048 | 1.08(0.58-2.01)0.794 | 3.28(0.50-21.52)0.210 |
| Q3 | 0.98(0.41-2.36)0.958 | 2.99(1.34-6.68)0.009 | 1.60(0.67-3.81)0.284 | 2.40(1.06-5.42)0.036 | 1.67(0.99-2.81)0.055 | 7.20(1.51-34.29)0.014 |
| Q4 | 1.44(0.55-3.79)0.446 | 1.84(0.83-4.08)0.131 | 1.20(0.44-3.27)0.716 | 2.41(0.98-5.91)0.054 | 1.60(0.81-3.15)0.168 | 2.00(0.21-18.75)0.533 |
| CYMA |  |  |  |  |  |  |
| Q1 | Reference | Reference | Reference | Reference | Reference | Reference |
| Q2 | 2.43(1.18-5.02)0.017 | 0.88(0.43-1.78)0.709 | 1.69(0.77-3.72)0.187 | 1.20(0.62-2.30)0.581 | 1.24(0.71-2.16)0.438 | 4.63(1.12-19.10)0.035 |
| Q3 | 3.12(1.41-6.86)0.006 | 1.81(0.79-4.13)0.155 | 2.66(1.20-5.91)0.017 | 2.02(1.03-3.95)0.040 | 2.56(1.44-4.54)0.002 | 1.07(0.25-4.55)0.926 |
| Q4 | 2.57(0.95-6.97)0.063 | 2.09(0.92-4.73)0.075 | 1.58(0.59-4.22)0.351 | 2.78(1.10-7.02)0.032 | 2.46(1.23-4.92)0.012 | 0.00(0.00-0.00)<0.001 |
| 2HPMA |  |  |  |  |  |  |
| Q1 | Reference | Reference | Reference | Reference | Reference | Reference |
| Q2 | 1.64(0.71-3.81)0.239 | 1.05(0.47-2.38)0.897 | 1.58(0.73-3.44)0.240 | 1.06(0.48-2.32)0.883 | 1.25(0.69-2.30)0.453 | 3.22(0.58-17.71)0.174 |
| Q3 | 1.82(0.82-4.06)0.138 | 1.35(0.54-3.42)0.514 | 1.94(0.90-4.20)0.090 | 1.28(0.64-2.56)0.476 | 1.60(0.89-2.89)0.114 | 4.42(1.17-16.74)0.030 |
| Q4 | 0.86(0.37-1.99)0.718 | 1.38(0.63-3.03)0.406 | 1.20(0.54-2.63)0.647 | 1.07(0.59-1.91)0.825 | 1.21(0.69-2.10)0.499 | 1.26(0.18-8.68)0.808 |
| 3HPMA |  |  |  |  |  |  |
| Q1 | Reference | Reference | Reference | Reference | Reference | Reference |
| Q2 | 1.30(0.60-2.81)0.500 | 0.99(0.41-2.37)0.984 | 1.39(0.54-3.62)0.486 | 1.09(0.46-2.57)0.837 | 1.19(0.64-2.21)0.580 | 1.45(0.39-5.42)0.570 |
| Q3 | 0.85(0.40-1.78)0.659 | 1.38(0.57-3.34)0.472 | 1.33(0.50-3.52)0.563 | 1.07(0.49-2.36)0.857 | 1.10(0.59-2.04)0.762 | 1.58(0.29-8.74)0.592 |
| Q4 | 1.51(0.54-4.22)0.418 | 1.22(0.51-2.93)0.649 | 1.61(0.67-3.92)0.282 | 1.54(0.70-3.38)0.271 | 1.58(0.82-3.05)0.165 | 0.43(0.06-2.91)0.381 |
| MA |  |  |  |  |  |  |
| Q1 | Reference | Reference | Reference | Reference | Reference | Reference |
| Q2 | 0.86(0.40-1.86)0.701 | 1.36(0.62-3.00)0.439 | 1.46(0.67-3.18)0.338 | 0.70(0.32-1.54)0.370 | 1.12(0.64-1.94)0.688 | 1.96(0.45-8.57)0.363 |
| Q3 | 1.32(0.59-2.92)0.489 | 1.12(0.45-2.80)0.806 | 1.22(0.54-2.76)0.626 | 1.49(0.65-3.41)0.342 | 1.31(0.71-2.43)0.378 | 2.10(0.30-14.64)0.443 |
| Q4 | 1.66(0.59-4.68)0.327 | 2.22(0.93-5.29)0.071 | 1.37(0.51-3.65)0.523 | 2.76(1.19-6.42)0.020 | 2.20(1.07-4.50)0.032 | 0.22(0.01-4.19)0.305 |
| MHBMA3 |  |  |  |  |  |  |
| Q1 | Reference | Reference | Reference | Reference | Reference | Reference |
| Q2 | 0.68(0.34-1.38)0.278 | 1.03(0.38-2.80)0.950 | 0.50(0.20-1.25)0.134 | 1.38(0.75-2.53)0.287 | 0.82(0.44-1.50)0.509 | 0.51(0.08-3.36)0.473 |
| Q3 | 0.87(0.36-2.14)0.759 | 1.65(0.75-3.65)0.209 | 0.71(0.28-1.80)0.463 | 2.00(1.09-3.66)0.025 | 1.11(0.57-2.14)0.761 | 3.71(0.49-28.33)0.201 |
| Q4 | 0.72(0.27-1.90)0.495 | 1.20(0.47-3.07)0.692 | 0.71(0.29-1.79)0.465 | 1.22(0.63-2.33)0.546 | 0.93(0.47-1.85)0.828 | 2.08(0.30-14.39)0.447 |
| PGA |  |  |  |  |  |  |
| Q1 | Reference | Reference | Reference | Reference | Reference | Reference |
| Q2 | 0.44(0.20-0.98)0.043 | 1.12(0.50-2.54)0.777 | 0.45(0.20-1.01)0.054 | 1.15(0.46-2.91)0.759 | 0.75(0.42-1.33)0.312 | 2.14(0.56-8.17)0.259 |
| Q3 | 1.45(0.68-3.09)0.321 | 1.11(0.43-2.85)0.830 | 0.77(0.36-1.61)0.476 | 2.14(0.81-5.64)0.119 | 1.23(0.69-2.18)0.474 | 7.35(1.11-48.63)0.039 |
| Q4 | 0.88(0.36-2.16)0.773 | 1.49(0.66-3.37)0.325 | 0.54(0.22-1.30)0.167 | 2.28(0.89-5.86)0.084 | 1.11(0.63-1.96)0.704 | 2.41(0.48-12.14)0.277 |
| HPMMA |  |  |  |  |  |  |
| Q1 | Reference | Reference | Reference | Reference | Reference | Reference |
| Q2 | 0.79(0.34-1.84)0.583 | 0.64(0.29-1.41)0.260 | 0.63(0.28-1.44)0.267 | 0.82(0.31-2.18)0.677 | 0.72(0.42-1.22)0.217 | 3.46(0.36-33.29)0.275 |
| Q3 | 1.24(0.59-2.61)0.568 | 1.45(0.59-3.55)0.411 | 0.95(0.40-2.27)0.912 | 2.07(0.96-4.46)0.062 | 1.28(0.73-2.26)0.378 | 7.58(0.72-80.18)0.090 |
| Q4 | 1.18(0.48-2.92)0.711 | 1.27(0.60-2.67)0.522 | 0.92(0.42-1.99)0.826 | 1.62(0.86-3.03)0.132 | 1.13(0.63-2.02)0.676 | 7.08(0.58-86.75)0.123 |

Supplementary Table S2: This table presents the odds ratios (OR) and 95% confidence intervals (CI) for the association between various mVOCs and the risk of sarcopenia, stratified by BMI (<25, ≥25), gender (male, female), and age (<40 years, ≥40 years). Significant associations (p < 0.05) are highlighted, and the logistic regression models were adjusted for age, sex, race/ethnicity, education level, poverty income ratio (PIR), smoking status, BMI, physical activity, diabetes, hypertension, alcohol consumption, and serum vitamin D levels.
