# Supplementary material for: Exposure to volatile organic compounds and sarcopenia risk in US adults based on NHANES
Source: Sci Rep. 2025 Jul 15;15:25480. doi: 10.1038/s41598-025-11628-0 (PMC12264073; doi:10.1038/s41598-025-11628-0)
Supplement: Supplementary file 2 — Supplementary Material 2 [file 41598_2025_11628_MOESM2_ESM.docx]

**Supplementary Table S1.** Relative parent volatile organic compounds (VOCs) to the metabolites of VOCs (mVOCs)

| Parent VOCs | Urine metabolites of VOCs (mVOCs) | Abbreviation of VOCs |
| --- | --- | --- |
| Acrolein | N-Acetyl-S-(2-carboxyethyl)-L-cysteine | CEMA |
|  | N-Acetyl-S-(2-hydroxypropyl)-L-cysteine | 2HPMA |
|  | N-Acetyl-S-(3-hydroxypropyl)-L-cysteine | 3HPMA |
| Acrylamide | N-Acetyl-S-(2-carbamoylethyl)-L-cysteine | AAMA |
| Acrylonitrile | N-Acetyl-S-(2-cyanoethyl)-L-cysteine | CYMA |
| Crotonaldehyde | N-Acetyl-S-(3-hydroxypropyl-1-methyl)-L-cysteine | HPMMA |
| Cyanide | 2-Aminothiazoline-4-carboxylic acid | ATCA |
| Ethylbenzene, styrene | Phenylglyoxylic acid | PGA |
|  | Mandelic acid | MA |
| N,N-Dimethylformamide | N-Acetyl-S-(N-methylcarbamoyl)-L-cysteine | AMCC |
| Propylene oxide | N-Acetyl-S-(2-hydroxypropyl)-L-cysteine | 2HPMA |
| Toluene | N-Acetyl-S-(benzyl)-L-cysteine | SBMA |
| Xylene | 2-Methylhippuric acid | 2MHA |
|  | 3- and 4-Methylhippuric acid | 3,4-MHA |
| 1,3-Butadiene | N-Acetyl-S-(4-hydroxy-2-butenyl)-L-cysteine | MHBMA3 |
