# Supplementary material for: Exposure to volatile organic compounds and sarcopenia risk in US adults based on NHANES
Source: Sci Rep. 2025 Jul 15;15:25480. doi: 10.1038/s41598-025-11628-0 (PMC12264073; doi:10.1038/s41598-025-11628-0)
Supplement: Supplementary file 3 — Supplementary Material 3 [file 41598_2025_11628_MOESM3_ESM.docx]

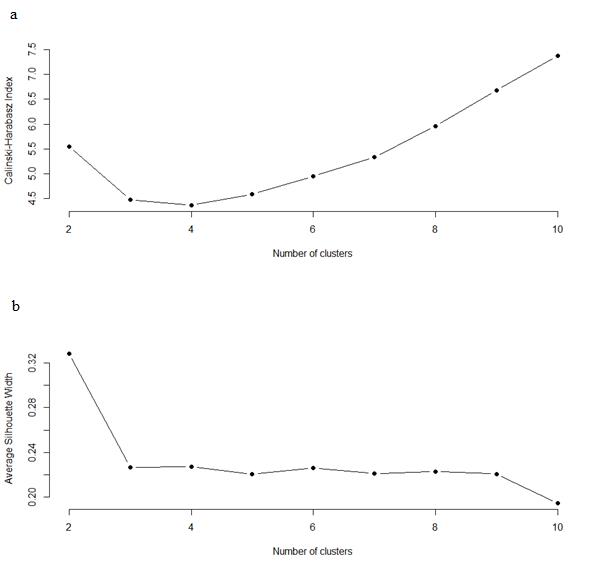


Supplementary Figure S1: Evaluation of the optimal number of clusters using the Calinski-Harabasz Index (a) and Silhouette Coefficient (b).
